# Latent Profile Analysis of Perceptions and Attitudes Towards COVID-19 in a Sample of Chinese People

**DOI:** 10.3389/fpubh.2021.727242

**Published:** 2021-09-27

**Authors:** Zhimin Niu, Li Li, Hongying Li, Songli Mei, Hui Jiang, Zhiyong Deng, Jun Xin

**Affiliations:** ^1^School of Humanities and Social Sciences, Gannan Medical University, Ganzhou, China; ^2^The First Affiliated Hospital, Soochow University, Suzhou, China; ^3^School of Public Health, Jilin University, Changchun, China; ^4^Zhangzhou Affiliated Hospital of Fujian Medical University, Zhangzhou, China; ^5^First People Hospital of Kunshan, Kunshan, China; ^6^Suzhou Municipal Party School, Suzhou, China

**Keywords:** perception, attitude, latent profile analysis, COVID-19, Chinese people

## Abstract

**Background:** The novel coronavirus-2019 (COVID-19) disease has spread quickly throughout China and around the world, endangering human health and life. Individuals' perceptions and attitudes as well as related health education measures may affect disease progression and prognosis during the COVID-19 outbreak. To promote and implement health education, research must focus on the perceptions and attitudes towards COVID-19 among Chinese people. The present study aimed to examine the profiles and predictive factors of the perceptions and attitudes towards COVID-19 in a sample of Chinese people.

**Methods:** A sample of 2,663 Chinese people comprising medical staff and members of the general public completed an online survey on Wenjuanxing. The survey measured demographic variables (e.g., gender, age, education level, and place of residence) and perceptions and attitudes towards COVID-19.

**Results:** Two profiles of perceptions and attitudes towards COVID-19 (positive and negative perceptions and attitudes) were identified in the sample. Place of residence during the COVID-19 pandemic and first response to COVID-19 were found to be independent predictive factors for COVID-19 related perceptions and attitudes.

**Conclusion:** In addition to drug therapy and mental health services, the perceptions and attitudes of Chinese people towards COVID-19 should be considered when promoting health education during the COVID-19 pandemic.

## Introduction

A new coronavirus (SARS-CoV-2) causes disease affecting the respiratory system and was named COVID-19 by the World Health Organization (WHO) (1). The novel COVID-19, with source of origin yet to be verified by the authorities, has spread rapidly from Wuhan in Hubei province to other cities in China and even all over the world since January 2020 (2). The WHO confirmed the global outbreak of COVID-19 in March 2020. A report from a French scholar indicated that COVID-19 was already spreading in France in late December 2019 (3). Some scientific evidence has indicated that COVID-19 originated in nature rather than being man-made in the laboratory (4–9). The WHO has stated that people aged 60 years and older are most at risk of severe illness from COVID-19. However, anyone can get sick from COVID-19 and become seriously and critically ill or experience complications (e.g., respiratory failure, acute respiratory distress syndrome [ARDS], and/or multiorgan failure) and even death. To date, there have been more than 80.7 million confirmed COVID-19 cases causing over 1.78 million deaths around the world (10). As an infectious disease with a high mortality rate, COVID-19 threatens the health and lives of all mankind.

The unclear aetiology of COVID-19 is a cause of worry and panic around the world. Cognitive-behavioural theories suggest that unreasonable perceptions and passive attitudes triggered by insufficient knowledge or other factors (e.g., social culture and family environment) may lead to negative emotions and dangerous behaviours in individuals, subsequently endangering their mental and physical health (11–13). Anyone who is infected risks passing on COVID-19 to others around him or her. However, some individuals continue partying and have close social contact, large meetings, and even gatherings during the COVID-19 pandemic, which may increase the COVID-19 infection and mortality rates. Therefore, the knowledges, perceptions, and attitudes towards COVID-19 of the general public and medical staff must be evaluated.

Recently, many scholars have focused on COVID-19 drug therapy (14–18) and mental health among different populations during the COVID-19 pandemic (19–25). However, some studies have focused on the knowledge and attitudes regarding COVID-19 among different populations by considering the previous responses to severe acute respiratory syndrome (SARS) (26–28), Middle East respiratory syndrome (MERS) and Asian lineage avian influenza A (H7N9) (29, 30). Research has investigated the knowledge and attitudes towards infectious diseases and the willingness to work during the COVID-19 outbreak among Chinese psychiatric hospital staff, and the findings suggest that most participants have extensive knowledge of COVID-19 (e.g., I understand how to protect myself and my patients) (31). Adequate knowledge and good attitudes regarding COVID-19 were also found among Pakistani university students and employees (i.e., pathogenesis, progression, prognosis and mortality of COVID-19) (32). Additionally, research exploring the attitudes towards mental health crisis services among clinically stable patients with COVID-19 has reported a positive attitude in half of the patients (33). Moreover, another survey of attitudes towards COVID-19 indicated that Chinese people were willing to comply with government guidelines on quarantine and social distancing during the COVID-19 outbreak (34).

The Chinese government adopted strict prevention and control measures to contain the COVID-19 outbreak from January 23, 2020 to April 8, 2020 (e.g., Wuhan lockdown, community closure management, quarantine, and restricted outdoor activities). Travel agencies and online travel companies suspended operations of most products and travel services all over China. Many urban and rural roads in China were blocked. Residents could not visit relatives and friends or attend gatherings. When they had to leave home to get food or work, they had to wear masks and undergo health checks by having their body temperature taken and having electronic health codes scanned on their smartphones (i.e., recording name, address and health condition) (35).

However, there have been few studies on the perceptions and attitudes towards COVID-19 among the Chinese general public and medical staff simultaneously, particularly when assessed with a latent profile analysis. Moreover, the lack of an approved cure for COVID-19 also requires that people have extensive knowledge for protecting their individual health and safety (e.g., maintain social distance, wear a face mask, and wash hands). Adequate knowledge and positive attitudes towards COVID-19 contribute to disease prevention and control. To better implement health education related to COVID-19 among Chinese people (i.e., residents in China), the aims of the present study were as follows: (i) to identify the profiles of perceptions and attitudes towards COVID-19 among Chinese people; (ii) to examine the differences in perceptions and attitudes towards COVID-19 between Chinese people based on demographic variables; and (iii) to examine the predictive factors for COVID-19 related perceptions and attitudes among Chinese people.

## Methods

The present study used a cross-sectional design and a convenience sample to assess the perceptions and attitudes towards COVID-19 using a self-report survey administered to a sample of 2663 Chinese people and examined the predictive factors for these perceptions and attitudes. Latent profile characteristics were also identified in this study.

### Participants

The sample comprised 805 males (30.2%) and 1858 females (69.8%) who were divided into three groups based on age: 18–45 years (2,073, 77.8%), 46–59 years (555, 20.9%), and older than 60 years (35, 1.3%). The education levels of the subjects were distributed as follows: 16.1% did not have an undergraduate degree, 52.7% had an undergraduate degree, and 31.2% had a post-graduate degree. The sample comprised 1339 medical staff (50.3%) and 1324 members of the general public (49.7%). Moreover, 2.5% lived in Hubei province whereas 97.5% lived in other provinces of China. A total of 13.4% had relatives or friends living in the COVID-19 epicentre in Hubei province.

### Measures

#### Demographic Data

Demographic data were collected by the Wechat platform and included gender (male or female), age (18–45 years, 46–59 years, or older than 60 years), education level (lower than undergraduate, postgraduate, or undergraduate), population (medical staff or general public), place of residence (Hubei province or other province), and having relatives or friends living in the COVID-19 epicentre in Hubei province (yes, no, or not sure).

#### Perceptions and Attitudes Towards COVID-19 Amongst Chinese People

Perceptions and attitudes towards COVID-19 were assessed using a questionnaire developed by the present authors. Three ethicists and three public health experts from China assessed the content validity of the questionnaire. Based on the Content Validity Index for Items (I-CVI) (36), the experts were asked to review the items referring to specific scenarios related to Perceptions and attitudes towards COVID-19. These items were assessed on a 4-point ordinal rating scale from 1 (‘*not relevant*’) to 4 (‘*highly relevant*’) and showed more than CVI of 0.78 by 6 experts. The actual CVI was a proportion of the items that received a rating of 3 or 4 by the experts. Finally, one item was excluded due to low I-CVI value (0.5) and the remaining items showed an intermediate S-CVI/UA (i.e., scale-level content validity index/universal agreement = 0.62). The questionnaire included 11 specific questions related to COVID-19 (Table 1). Four items assessed the awareness of COVID-19, including the outbreak time and duration of the pandemic, the severity of COVID-19, and the frequency of mask replacement (e.g., ‘*When did you first know about the COVID-19 pandemic?’*). The answer options included before December 28, 2019 (i.e., ‘Initial report: Unexplained pneumonia cases were reported to Health Commissions of Wuhan city and Hubei province by a doctor named Zhang Jixian’), before January 17, 2020 (i.e., ‘Findings of an epidemiological investigation: Evidence of person-to-person transmission and infection without symptoms were found by an academic group from Hong Kong University’), before January 20, 2020 (i.e., ‘Supreme command: The top leader of China required the Wuhan government to take effective measures and control the outbreak’), or after January 23, 2020 (i.e., ‘Hubei province government announced Wuhan lockdown’) (37). Furthermore, reactions to the COVID-19 pandemic were assessed by two items (e.g., ‘*When you learned that the COVID-19 pandemic was serious, what was your first reaction?*’). The last five items were related to perceptions and attitudes towards COVID-19 (e.g., ‘*At 10 o’clock in the morning of January 23, 2020, the city of Wuhan was closed. What do you think about this measure?’*) and were rated on a four-point Likert scale ranging from 1 (*disagree*) to 4 (*agree*); the total score ranged from 5 to 20. Higher scores represented more positive perceptions and attitudes towards COVID-19. The 5-item model fit the data well in the principal component analysis (PCA) (the component loadings were ranged between 0.4 and 0.6 for items 7–11, respectively; χ^2^ = 191, *df* = 10).

**Table 1:** Questions and answer options regarding perceptions and attitudes towards COVID-19.

	**Questions**	**1**	**2**	**3**	**4**
	**Awareness of COVID-19**				
1	When did you first know about the COVID-19 pandemic?	before December 28, 2019	before January 17, 2020	before January 20, 2020	After January 23, 2020
2	When you first learned of the COVID-19 outbreak, how serious did you think it was?	not sure	mild	moderate	severe
3	How often do you think masks worn by the general public must be replaced?	every 3 days	every day	every half day	it depends
4	Some experts think the COVID-19 pandemic may continue until February 8, 2020. What do you think?	not sure	February 8	the end of February	the end of May
	**Response towards COVID-19**				
5	When you learned that the COVID-19 pandemic was serious, what was your first reaction?	do nothing	inform relatives and friends	purchase life supplies	fight against COVID-19
6	What do you do when you go out to take the subway or bus without wearing a mask and are reprimanded by the cabin crew or others?	walk or change transportation	cover your nose and mouth with your sleeves	ignore	it depends
	**perceptions and attitudes towards COVID-19**				
7	On January 22, a man visited his relatives in Wuhan when the city closed. Afraid that the closure would affect his work, he drove his wife and children to the home of a certain city community on the 23rd and was stopped by community security. What do you think about this measure?	disagree	not sure	somewhat agree	agree
8	At 10 o'clock in the morning of January 23, 2020, the city of Wuhan was closed. What do you think about this measure?	disagree	not sure	somewhat agree	agree
9	Should you send your family members to the hospital if they have a fever and cough during the COVID-19 pandemic? What do you think?	disagree	not sure	somewhat agree	agree
10	To quickly curb the pandemic in Wuhan, the country adopted a unified arrangement requiring people who had visited or had contact with people in key pandemic areas in Hubei to go to designated hotels for medical isolation after January 10, 2020. What do you think about this measure?	disagree	not sure	somewhat agree	agree
11	Two patients with severe COVID-19 pneumonia were in urgent need of hospitalization at the same time in the emergency department. One was a 36-year-old respiratory nurse in a hospital and the other was a 35-year-old cadre who had assisted Tibet and had just returned to town. A bed can be vacated. If you are a doctor on duty, will you save the Tibetan cadre first?	disagree	not sure	somewhat agree	agree

### Procedure

The First Affiliated Hospital of Soochow University and First People Hospital of Kunshan were responsible for collecting the related data. The sample, which included medical staff and members of the general public, was recruited through several WeChat groups (medicine, ethics, health management and related communities) with different occupations (e.g., medical staff, students, teachers, civil servants, and freelancers) who lived in different provinces of China (e.g., Jiangsu, Hubei, Jiangxi, Henan, Shanxi, Fujian, and Zhejiang). The survey was conducted from January 28, 2020, to February 4, 2020. The participants completed the online survey on the Wenjuanxing platform (www.wjx.cn). The survey was completed in ~5 min.

### Statistical Analysis

To explore the most likely number of classes based on the 5-item perceptions and attitudes questionnaire, a latent profile analysis (LPA) was performed using Mplus 7.0. The participants were aggregated into two to five groups to test the latent profiles. The best-fit statistics and interpretability were considered criteria for the optimal model (38). Model fit was assessed by several statistical indices, including the Akaike information criterion (AIC), the Bayesian information criterion (BIC), the sample size-adjusted BIC (A-BIC), the bootstrap likelihood ratio test (BLRT) and the Lo-Mendell-Rubin adjusted likelihood ratio test (LMRA-LRT). Values exceeding 0.8 and approaching 1.0, as well as decreased AIC, BIC, and A-BIC values, indicated much clearer results (39). The LMRA-LRT and BLRT with low and significant *p*-values signified a better-fitting model (40). Descriptive statistics, Fisher's exact test, and *post hoc* testing (i.e., an adjusted standardised residual with a value greater than 3 is considered to indicate a significant difference between observed and expected values) (41) were performed using SPSS 20. A PCA was conducted to assess perceptions and attitudes towards COVID-19 using Jamovi. A logistic stepwise regression was also performed using Jamovi with the probability of entry as 0.05, which may predict independent factors for the perceptions and attitudes towards COVID-19.

### Ethics

This study was reviewed and approved by the Ethics Committee of Soochow University and was conducted in accordance with the ethical standards in the Declaration of Helsinki. Informed consent was signed by all participants after reading a statement of the study's purpose, procedure and confidentiality.

## Results

### Descriptive Characteristics of the Participants

As shown in Table 2, Chi-square tests revealed that males had demonstrated statistically significantly more objective than females in the perceptions and attitudes towards COVID-19 (all *p* < 0.01). Similarly, medical staff had shown statistically significantly more positive than the general public in the perceptions and attitudes towards COVID-19 (all *p* < 0.01). Additionally, no significant differences in the awareness, reaction, perceptions and attitude towards COVID-19 were noted between the subjects with different places of residence (Hubei and other places) (*p* > 0.05 for most items; Appendix S1 in Supplementary Material). Statistically, the lower age group showed significantly quicker reaction than the middle age group in response to COVID-19 (absolute value of adjusted residuals >3), whilst the higher educational level performed significantly more objectively in the perceptions and attitudes towards COVID-19 (absolute value of adjusted residuals >3; Appendix S2 in Supplementary Material).

**Table 2:** Demographic characteristics of participants (*N* = 2,263).

**Variables**	**Gender**		**Population**		**Place of residence**		**Age**		**Education level**		**Relatives or friends living in COVID-19 epicentre**	
	***X^2^***	***p***	***X^2^***	***p***	***X^2^***	***p***	***X^2^***	***p***	***X^2^***	***p***	***X^2^***	***p***
**Awareness**												
1	14.02	0.003	18.19	< 0.001	14.96	0.001	25.33	< 0.001	74.26	< 0.001	11.36	0.073
2	2.64	0.451	51.66	< 0.001	4.66	0.195	16.69	0.010	44.21	< 0.001	15.62	0.014
3	40.72	< 0.001	34.99	< 0.001	0.48	0.933	19.46	0.003	24.76	< 0.001	7.78	0.239
4	56.23	< 0.001	5.60	0.129	5.38	0.143	76.57	< 0.001	96.25	< 0.001	32.88	< 0.001
**Reaction**												
5	88.29	< 0.001	71.98	< 0.001	23.51	< 0.001	136.91	< 0.001	42.15	< 0.001	9.40	0.140
6	4.67	0.195	26.07	< 0.001	4.71	0.155	51.62	< 0.001	20.43	0.002	30.22	< 0.001
**Perceptions and attitudes**												
7	98.32	< 0.001	7.18	0.062	4.12	0.195	22.12	0.001	68.29	< 0.001	32.58	< 0.001
8	14.76	0.002	9.99	0.018	13.08	0.004	26.77	< 0.001	28.12	< 0.001	12.11	0.048
9	37.56	< 0.001	6.07	0.103	7.41	0.050	47.80	< 0.001	78.06	< 0.001	11.41	0.057
10	17.64	< 0.001	13.88	0.003	17.89	< 0.001	2.30	0.826	17.60	0.005	34.18	< 0.001
11	56.21	< 0.001	44.75	< 0.001	6.96	0.063	7.53	0.268	159.31	< 0.001	27.16	< 0.001

*For items 1–11, see Table 1. Fisher's exact test was used in the analysis of sizes (<5). Population: medical staff and general public. Place of residence: Hubei and others. Age (years): 18-45, 46–59, and ≥60. Education level: lower than undergraduate, undergraduate, and postgraduate. Relatives or friends living in COVID-19 epicentre: Yes, No, and Not sure*.

### Latent Profile Analysis

**Figure 1:**
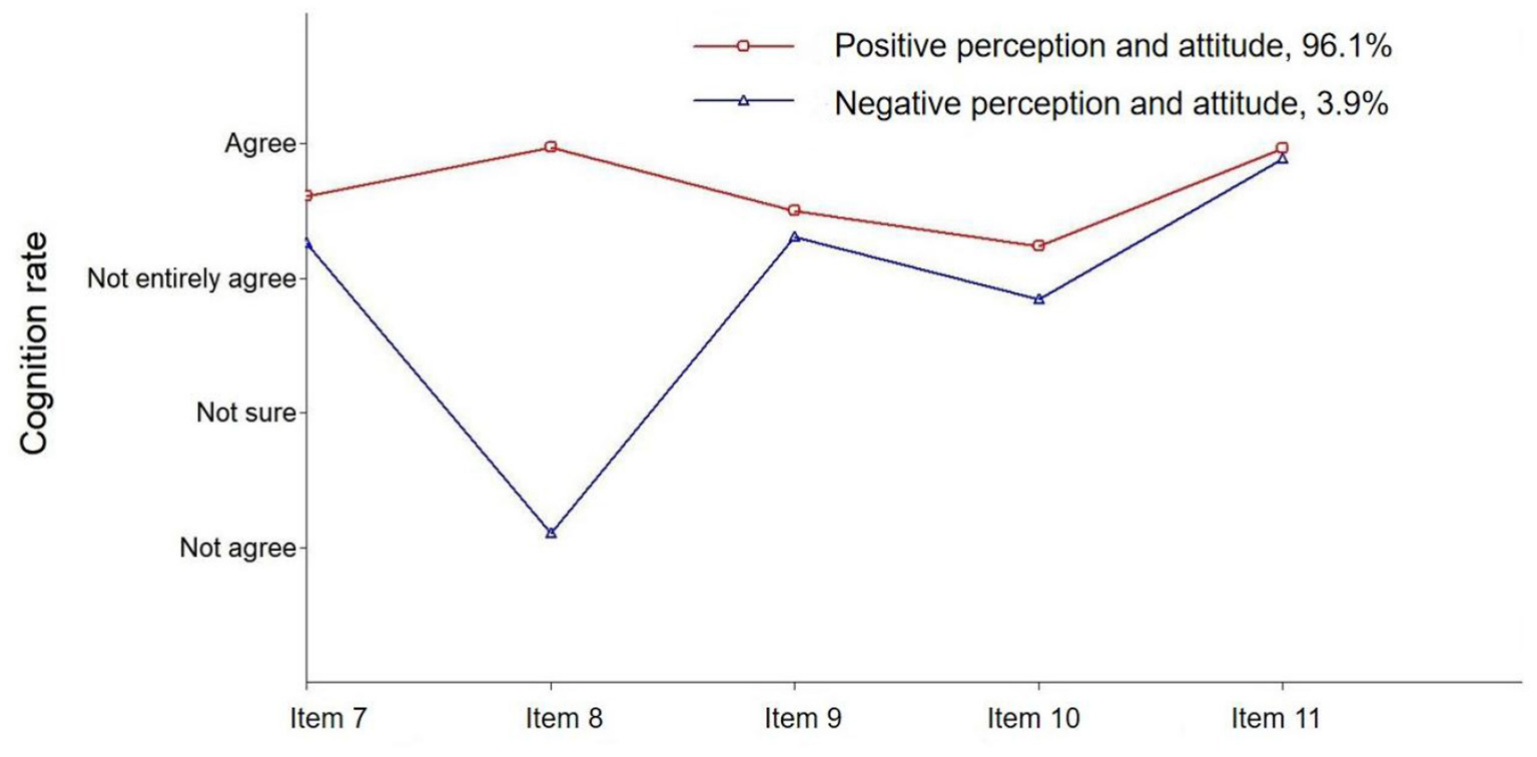
Latent class profile related to the perceptions and attitudes towards COVID-19.

Four models with two to five subgroups were tested, and the fit indices are shown in Table 3. The AIC, BIC and A-BIC values decreased gradually from the two-profile to the five-profile models. The entropy values for the different profiles ranged from 0.999 to 1.000. However, only the two-profile LMR-LTR were less than 0.001, whereas the other profiles had no significant LMR-LTR *p*-values. Additionally, the theoretical meaningfulness of perceptions and attitudes was considered to determine the best profile. Two simple profiles of perceptions and attitudes towards COVID-19 were identified for the sample of 2,663 Chinese people: (i) the positive perceptions and attitudes group (*n* = 2,559, 96.1%) and (ii) the negative perceptions and attitudes group (*n* = 104, 3.9%; Figure 1).

**Table 3:** Fit indices for the latent profile analysis of 5 items on perceptions and attitudes towards COVID-19.

**Model**	***k***	**AIC**	**BIC**	**A-BIC**	**Entropy**	**LMR-LTR** **(***p***)**	**BLRT** **(***p***)**
Class 2	16	17407.261	17501.456	17450.619	1.000	**<0.001**	<0.001
Class 3	22	12175.290	12304.809	12234.908	1.000	0.5176	<0.001
Class 4	28	9420.092	9584.934	9495.970	1.000	0.6374	<0.001
Class 5	34	8279.965	8480.130	8372.102	0.999	0.0940	<0.001

*AIC, Akaike Information Criterion; BIC, Bayesian Information Criterion; A-BIC, Sample Size-Adjusted BIC; LMR-LRT, Lo-Mendell Rubin Likelihood Ratio Test; BLRT, Bootstrap Likelihood Ratio Test. LMRA-LRT with a significant p-value signified a better-fitting model*.

### Logistic Stepwise Regression of Perceptions and Attitudes Towards COVID-19

A logistic stepwise regression analysis was used to assess the independent predictive factors of perceptions and attitudes towards COVID-19. In the univariate analysis, place of residence, mask replacement, and first response to COVID-19 were predictive factors for the perceptions and attitudes towards COVID-19. In the multivariate analysis, place of residence and first response to COVID-19 were found to be independent predictive factors for the perceptions and attitudes towards COVID-19 (Table 4).

**Table 4:** Logistic stepwise regression of perceptions and attitudes towards COVID-19.

**Variables**	**OR (95% CI)**	**Single factor**		**OR (95% CI)**	**Multiple factors**	
		***B (SE)***	***p***		***B (SE)***	***p***
Gender	1.02 (0.67, 1.57)	0.02 (0.22)	0.924			
Age	0.66 (0.40, 1.09)	−0.42 (0.26)	0.105			
Education level	1.27 (0.94, 1.70)	0.24 (0.15)	0.121			
Occupation	0.95 (0.83, 1.09)	−0.05 (0.07)	0.480			
Place of residence	0.40 (0.17, 0.95)	−0.92 (0.44)	**0.037**	0.38 (0.16, 0.90)	−0.98 (0.44)	**0.028**
Relatives	0.65 (0.40, 1.05)	−0.44 (0.25)	0.078			
Item 1	1.04 (0.84, 1.27)	0.03 (0.11)	0.746			
Item 2	0.90 (0.74, 1.10)	−0.11 (0.10)	0.301			
Item 3	0.70 (0.50, 0.99)	−0.35 (0.18)	**0.044**	0.71 (0.51, 1.01)	−0.34 (0.18)	0.055
Item 4	0.96 (0.70, 1.33)	−0.04 (0.17)	0.817			
Item 5	0.72 (0.58, 0.89)	−0.33 (0.11)	**0.002**	0.72 (0.58, 0.89)	−0.33 (0.11)	**0.003**
Item 6	0.92 (0.76, 1.10)	−0.09 (0.09)	0.355			

*OR, Odds Ratio; CI, Confidence Interval. For items 1–6 see Table 1. p < 0.05 was considered to indicate statistical significance*.

## Discussion

The present study examined the perceptions and attitudes towards COVID-19 using a self-reported questionnaire among a sample of 2663 Chinese people. Due to the unexpected and abrupt nature of the COVID-19 outbreak, no proven treatment or vaccination against the coronavirus was available. Public awareness of dealing with infectious diseases may play a vital role in struggling with COVID-19. Investigations on perceptions and attitudes towards COVID-19 are extremely urgent and essential (42). Therefore, some scenarios that are specifically related to COVID-19 (e.g., lockdown, health and security checks, and medical isolation during COVID-19, which are unexpected and subject to frequent repetitions) were examined in the questionnaire.

The present study examined the differences in the perceptions and attitudes towards COVID-19 between subjects based on demographic variables, profiles and predictive factors of perceptions and attitudes towards COVID-19 in a sample of 2663 Chinese people. There were statistically significant differences in the perceptions and attitudes towards COVID-19 based on gender. Male participants reported a more objective perceptions and attitudes towards COVID-19 than females (i.e., more males provided the answer ‘disagree’ than females, from item 7 to item 11 *p* < 0.01). These results may be explained by the stereotypes of sex differences. These stereotypes suggest that males are good at thinking rationally and timely and take practical actions to solve problems, whereas females are more emotional (43, 44). For example, some males prefer to analyse issues using reasonable logic rather than intuitive thinking. Additionally, medical staff who have more professional medical knowledge, skills and professional ethics showed more objective perceptions and attitudes towards COVID-19 than the general public (i.e., items 10 and 11 *p* < 0.01). One study reported that Chinese medical staff have rich medical knowledge, have completed at least 3 or 4 years of vocational training (e.g., nurse and medical technician), and understand the risks of various diseases (31).

Younger participants (18–45 years) preferred to inform others and fought less against COVID-19 than the middle age group (46–59 years) (i.e., items 5 and 6 absolute value of adjusted residuals > 3). A possible explanation for this finding is the lack of work experience in younger individuals. Young people have a high level of enthusiasm and willing to help others but have less work experience compared with older individuals. In addition, participants with a higher education level had relatively objective perceptions and attitudes towards COVID-19 (i.e., items 7, 9, and 11 absolute value of adjusted residuals > 3), which is consistent with their educational background. These participants give relatively rational answers (e.g., somewhat agree or not sure) instead of ‘fully agree’, which may be consistent with the process of knowledge and discovery and indicates the importance of knowledge. This finding suggests that it is important for health regulators to implement health education programs towards COVID-19 as soon as possible.

Latent profile analysis is useful to distinguish perceptions and attitudes towards COVID-19 among Chinese people, and data from such analyses can be used to implement sound health education and public health prevention. Two profiles of perceptions and attitudes towards COVID-19 (i.e., the positive perceptions and attitudes group and the negative perceptions and attitudes group) were found in this sample of 2,663 Chinese people. The results indicated that greater than 96% of the participants had positive perceptions and attitudes towards COVID-19. Participants agreed that they should participate in effective measures against COVID-19, including medical isolation, city lockdowns, community security, and health prevention. Moreover, the positive perceptions and attitudes towards COVID-19 also included altruism in the process of treatment (i.e., Tibetan cadre will be given priority for treatment because they worked voluntarily in Tibet under difficult climate conditions). The participants believed that the government and the people could win the battle against the COVID-19 pandemic, similar to the fight against SARS (45, 46), The results were also consistent with the progression of the COVID-19 pandemic in China, which declined significantly at the end of February (47). Although less than 4% of participants have negative perceptions and attitudes towards COVID-19, health education remains necessary and effective intervention against COVID-19. As an important health education measure, public communication by the government and society has yielded good results during the COVID-19 outbreak (48).

In early December 2019, some media began to pay attention to an unexplained pneumonia in Wuhan, referring in particular to the South China seafood market (49). On December 31, 2019, the Health Commission of Hubei Province first announced a cluster of unexplained cases of pneumonia (50). In the next 2 weeks, the initially reported number of 27 patients was revised to 41, with seven severe cases and one death (51). Then, evidence of person-to-person transmission and infection without symptoms was found on January 17, 2020 (52). On January 20, an academician of the Chinese Academy of Engineering named Zhong Nanshan affirmed that COVID-19 can be transmitted from person to person (53). Subsequently, Wuhan was closed at 2 a.m. on January 23, 2020. At this time, people all over China began to focus on COVID-19, and most Chinese people, except frontline staff and patients, quarantined at home. The negative perceptions and attitudes towards COVID-19 may lead to negative emotions (e.g., fear, panic, anxiety, and depression) and worsen mental and physical health, even causing public panic and an unstable society.

The perceptions and attitudes towards COVID-19 must be investigated and understood to better implement health education regarding the COVID-19 pandemic. Thus, a logistic stepwise regression was performed in the present study. The results of the single-factor analysis indicated that place of residence, frequency of mask replacement, and first response to COVID-19 were predictive factors for perceptions and attitudes towards COVID-19. Subsequently, multivariate logistic regression indicated that place of residence and first response to COVID-19 were independent predictive factors for perceptions and attitudes towards COVID-19. Participants who lived in the COVID-19 epicentre (i.e., Hubei) experienced more panic and anxiety, and these individuals were more likely to generate negative perceptions and attitudes towards COVID-19 when they faced life threatening scenarios due to this infectious disease. Regarding initial responses to COVID-19, passive people do nothing and passively waiting for the misfortune, whereas optimistic people will positively cope with COVID-19 and build positive perceptions and attitudes towards COVID-19. Based on the theory of reasoned action (TRA) (54), behavioural intention may be predicted through individual attitude and perception and can lead to different consequences. A positive attitude and perception are conducive to winning the battle against the COVID-19 pandemic in China and in Chinese communities abroad (55). City lockdowns, stringent quarantines, and local public health measures imposed from late January to late February significantly decreased COVID-19 transmission and infection (56). Moreover, knowledge and certain risk factors, including work experience and job category, impacted health care workers’ attitudes and practises related to COVID-19 (57).

Two profiles of perceptions and attitudes (positive and negative) towards COVID-19 were identified among Chinese people in the present study. Nevertheless, the present study has some limitations. Firstly, a single self-report was applied, and some items did not comply with the criteria of the Likert scale. In addition, items related to perceptions and attitudes were rated on a four-point Likert scale, including ‘*disagree’*, ‘*not sure’, ‘somewhat agree’*, and ‘*agree*’, which may cause a biassed result. The items regarding scenario-specific perceptions and attitudes towards COVID-19 may also partly indicate that the public trusts and supports government-implemented measures. Therefore, rigorous psychometric methods, including item analysis and reliability and validity analyses, were not available for this investigation of the perceptions and attitudes towards COVID-19. Secondly, all data were collected from a convenience sample by Wenjuanxing, which may also have led to selection biases, including social desirability, individual motivation, and relatively large proportion of females, thus limiting the sample's representativeness of the population. Retrospective studies and implicit measurements using representative random samples should be conducted in future studies. Thirdly, the impact of health education on the perceptions and attitudes towards COVID-19 should be delineated in future research. Finally, the relationship between perceptions and attitudes towards COVID-19 and risk perception and behavioural intervention for survivors of different ages, countries, and populations (e.g., medical staff and the general public) should be explored, and longitudinal studies should be conducted.

## Conclusion

Overall, the present study has identified two profiles of perceptions and attitudes towards COVID-19 for a sample of Chinese people, which indicates that a positive perceptions and attitudes are helpful for winning the battle against COVID-19. Distorted cognition may cause negative emotions (e.g., fear, panic, anxiety and depression) and risky behaviours (e.g., partying during the COVID-19 pandemic, substance addiction or behavioural addiction), which may influence an individual's psychological and physical health. There is a clear need for campaigns to increase training programs providing public health education, which may help to distribute extensive knowledge of disease prevention, promote public health perception, and shape positive and optimistic attitudes towards COVID-19 amongst the Chinese people and even people around the world. Therefore, health authorities should take more effective measures to implement health training and education (e.g., health advocacy by media tools including TV, smartphone, and social networking platforms) to cope with the COVID-19 pandemic.

## Data Availability Statement

The original contributions presented in the study are included in the article/Supplementary Material, further inquiries can be directed to the corresponding author.

## Ethics Statement

The studies involving human participants were reviewed and approved by the Ethics Committee of Soochow University. The patients/participants provided their written informed consent to participate in this study.

## Author Contributions

HL and ZN conceived and designed the experiments. HL, HJ, ZD, JX, and ZN performed the experiments. LL analysed and interpreted the data. SM contributed reagents, materials, and analysis tools. ZN and LL wrote the first draft of the manuscript. All authors contributed to the article and approved the submitted version.

## Funding

The Science Education Program Project ‘Thirteenth 5-Year Plan’ of Jiangxi Province 2020GX184, Doctor start-up fund of Gannan Medical University QD201819, Key project of Gannan Medical University ZD201838, and The International Innovation Team of Jilin University 2019GJTD06.

## Conflict of Interest

The authors declare that the research was conducted in the absence of any commercial or financial relationships that could be construed as a potential conflict of interest.

## Publisher's Note

All claims expressed in this article are solely those of the authors and do not necessarily represent those of their affiliated organizations, or those of the publisher, the editors and the reviewers. Any product that may be evaluated in this article, or claim that may be made by its manufacturer, is not guaranteed or endorsed by the publisher.
