# Supplementary material for: Latent Profile Analysis of Perceptions and Attitudes Towards COVID-19 in a Sample of Chinese People
Source: Front Public Health. 2021 Sep 27;9:727242. doi: 10.3389/fpubh.2021.727242 (PMC8502915; doi:10.3389/fpubh.2021.727242)
Supplement: Supplementary file 1 [file Table_1.DOC]

**Supplementary materials**

**Appendix S1**. Demographic characteristics of participants (N=2263)

| Variables | All participants  (N=2663) | Gender | | *p* | Population | | *p* | Place of residence | | *p* |
| --- | --- | --- | --- | --- | --- | --- | --- | --- | --- | --- |
|  |  | Male  (N=805) | Female  (N=1858) | <0.001 | Medical staff  (N=1339) | General public  (N=1324) |  | Hubei  (N=67) | Others  (N=2596) |  |
| **Awareness of COVID-19 (items 1-4)** |  |  |  |  |  |  |  |  |  |  |
| 1 |  |  |  | 0.003 |  |  | <0.001 |  |  | 0.001 |
| Before Dec 28^th^, 2019 | 718 (27%) | 243 (30.2%) | 475 (25.6%) |  | 347 (25.9%) | 371 (28%) |  | 28 (41.8%) | 690 (26.6%) |  |
| Before Jan 17^th^, 2020 | 852 (32%) | 260 (32.3%) | 592 (31.9%) |  | 446 (33.3%) | 406 (30.8%) |  | 26 (38.8%) | 826 (31.8%) |  |
| Before Jan 20^th^, 2020 | 855 (32.1%) | 224 (27.8%) | 631 (34%) |  | 450 (33.7%) | 405 (30.7%) |  | 11 (16.4%) | 844 (32.5%) |  |
| Before Jan 23^rd^, 2020 | 238 (8.9%) | 78 (9.7%) | 160 (8.5%) |  | 96 (7.1%) | 142 (10.7%) |  | 2 (3%) | 236 (9.1%) |  |
| 2 |  |  |  | 0.451 |  |  | <0.001 |  |  | 0.195 |
| Not sure | 255 (9.6%) | 68 (8.4%) | 187 (10.1%) |  | 97 (7.2%) | 158 (11.9%) |  | 7 (10.4%) | 248 (9.6%) |  |
| Mild | 666 (25%) | 211 (26.2%) | 455 (24.5%) |  | 292 (21.8%) | 374 (28.2%) |  | 24 (35.8%) | 642 (24.7%) |  |
| Moderate | 926 (34.8%) | 277 (34.4%) | 649 (34.9%) |  | 501 (37.4%) | 425 (32.1%) |  | 20 (29.9%) | 906 (34.9%) |  |
| Severe | 816 (30.6%) | 249 (31%) | 567 (30.5%) |  | 449 (33.6%) | 367 (27.8%) |  | 16 (23.9%) | 800 (30.8%) |  |
| 3 |  |  |  | <0.001 |  |  | <0.001 |  |  | 0.933 |
| Every 3 days | 101 (3.8%) | 54 (6.7%) | 47 (2.5%) |  | 41 (3.1%) | 60 (4.5%) |  | 2 (3%) | 99 (3.8%) |  |
| Every day | 774 (29.1%) | 241 (29.9%) | 533 (28.7%) |  | 446 (33.3%) | 328 (24.8%) |  | 32 (47.8%) | 752 (29%) |  |
| Every half day | 655 (24.6%) | 211 (26.2%) | 444 (23.9%) |  | 304 (22.7%) | 351 (26.5%) |  | 15 (22.4%) | 640 (24.7%) |  |
| It depends | 1133 (42.5%) | 299 (37.2%) | 834 (44.9%) |  | 548 (40.9%) | 585 (44.2%) |  | 28 (41.8%) | 1105 (42.5%) |  |
| 4 |  |  |  | <0.001 |  |  | 0.129 |  |  | 0.143 |
| Not sure | 746 (28%) | 157 (19.5%) | 589 (31.7%) |  | 398 (29.7%) | 348 (26.3%) |  | 12 (17.8%) | 734 (28.3%) |  |
| Feb 8^th^ | 266 (10%) | 90 (11.2%) | 176 (9.5%) |  | 132 (9.9%) | 134 (10.1%) |  | 6 (9%) | 260 (10%) |  |
| The end of February | 1229 (46.2%) | 397 (49.3%) | 832 (44.8%) |  | 598 (44.7%) | 631 (47.7%) |  | 40 (59.7%) | 1189 (45.8%) |  |
| The end of May | 422 (15.8%) | 161 (20%) | 261 (14%) |  | 211 (15.7%) | 211 (15.9%) |  | 9 (13.5%) | 413 (15.9%) |  |
| **Reaction towards COVID-19 (items 5-6)** |  |  |  |  |  |  |  |  |  |  |
| 5 |  |  |  | <0.001 |  |  | <0.001 |  |  | <0.001 |
| Do nothing | 168 (6.3%) | 99 (12.3%) | 69 (3.7%) |  | 53 (3.9%) | 115 (8.7%) |  | 6 (9%) | 162 (6.2%) |  |
| Inform others | 1916 (71.9%) | 533 (66.2%) | 1383 (74.5%) |  | 1013 (75.6%) | 903 (68.2%) |  | 36 (53.7%) | 1880 (72.4%) |  |
| Prepare supplies | 433 (16.3%) | 117 (14.5%) | 316 (17%) |  | 179 (13.4%) | 254 (19.2%) |  | 25 (37.3%) | 408 (15.7%) |  |
| Fight against COVID-19 | 146 (5.5%) | 56 (7%) | 90 (4.8%) |  | 94 (7.1%) | 52 (3.9%) |  | 0 (0%) | 146 (5.7%) |  |
| 6 |  |  |  | 0.195 |  |  | <0.001 |  |  | 0.155 |
| Walk or change transportation | 25 (0.9%) | 7 (0.9%) | 18 (1%) |  | 8 (0.6%) | 17 (1.3%) |  | 1 (1.5%) | 24 (0.9%) |  |
| Cover your nose and mouth with your sleeves | 114 (4.3%) | 40 (5%) | 74 (4%) |  | 36 (2.7%) | 78 (5.9%) |  | 3 (4.5%) | 111 (4.3%) |  |
| Ignore | 1505 (56.5%) | 434 (53.9%) | 1071 (57.6%) |  | 774 (67.8%) | 731 (55.2%) |  | 45 (67.2%) | 1460 (56.2%) |  |
| It depends | 1019 (38.3%) | 324 (40.2%) | 695 (37.4%) |  | 521 (38.9%) | 498 (37.6%) |  | 18 (26.8%) | 1001 (38.6%) |  |
| **Perception and attitude towards COVID-19 (items 7-11)** |  |  |  |  |  |  |  |  |  |  |
| 7 |  |  |  | <0.001 |  |  | 0.062 |  |  | 0.195 |
| Disagree | 16(0.6%) | 8 (1%) | 8 (0.4%) |  | 5 (0.4%) | 11 (0.8%) |  | 0 (0%) | 16 (0.6%) |  |
| Not sure | 38(1.4%) | 13 (1.6%) | 25 (1.3%) |  | 14 (1%) | 24 (1.8%) |  | 2 (3%) | 36 (1.4%) |  |
| Somewhat agree | 924(34.7%) | 277 (34.4%) | 647 (34.8%) |  | 457 (34.2%) | 467 (35.3%) |  | 29 (43.3%) | 895 (34.5%) |  |
| Agree | 1685(63.3%) | 507 (63%) | 1178 (63.5%) |  | 863 (64.4%) | 822 (62.1%) |  | 36 (53.7%) | 1649 (63.5%) |  |
| 8 |  |  |  | 0.002 |  |  | 0.018 |  |  | 0.004 |
| Disagree | 11(0.4%) | 5 (0.6%) | 6 (0.3%) |  | 5 (0.4%) | 6 (0.5%) |  | 1 (1.5%) | 10 (0.4%) |  |
| Not sure | 93(3.5%) | 26 (3.2%) | 67 (3.6%) |  | 51 (3.8%) | 42 (3.2%) |  | 5 (7.5%) | 88 (3.4%) |  |
| Somewhat agree | 81(3%) | 38 (4.7%) | 43 (2.3%) |  | 29 (2.2%) | 52 (3.9%) |  | 6 (9%) | 75 (2.9%) |  |
| Agree | 2478(93.1%) | 736 (91.5%) | 1742 (93.8%) |  | 1254 (93.6%) | 1224 (92.4%) |  | 55 (82.%) | 2423 (93.3%) |  |
| 9 |  |  |  | <0.001 |  |  | 0.103 |  |  | 0.050 |
| Disagree | 77(2.9%) | 25 (3.1%) | 52 (2.8%) |  | 40 (3%) | 37 (2.8%) |  | 4 (6%) | 73 (2.8%) |  |
| Not sure | 11(0.4%) | 8 (1%) | 3 (0.2%) |  | 2 (0.1%) | 9 (0.7%) |  | 0 (0%) | 11 (0.4%) |  |
| Somewhat agree | 1161(43.6%) | 294 (36.5%) | 867 (46.7%) |  | 576 (43%) | 585 (44.2%) |  | 37 (55.2%) | 1124 (43.3%) |  |
| Agree | 1414(53.1%) | 478 (59.4%) | 936 (50.3%) |  | 721 (53.9%) | 693 (52.3%) |  | 26 (38.8%) | 1388 (53.5%) |  |
| 10 |  |  |  | <0.001 |  |  | 0.003 |  |  | <0.001 |
| Disagree | 11(0.4%) | 8 (1%) | 3 (0.2%) |  | 1 (0.1%) | 10 (0.8%) |  | 3 (4.5%) | 8 (0.3%) |  |
| Not sure | 18(0.7%) | 8 (1%) | 10 (0.5%) |  | 6 (0.5%) | 12 (0.9%) |  | 2 (3%) | 16 (0.6%) |  |
| Somewhat agree | 29(1.1%) | 13 (1.6%) | 16 (0.9%) |  | 18 (1.3%) | 11 (0.8%) |  | 1 (1.5%) | 28 (1.1%) |  |
| Agree | 2605(97.8%) | 776 (96.4%) | 1829 (98.4%) |  | 1314 (98.1%) | 1291 (97.5%) |  | 61 (91%) | 2544 (98%) |  |
| 11 |  |  |  | <0.001 |  |  | <0.001 |  |  | 0.063 |
| Disagree | 279(10.5%) | 156 (19.4%) | 202 (10.9%) |  | 156 (11.6%) | 202 (15.3%) |  | 16 (23.9%) | 342 (13.2%) |  |
| Not sure | 358(13.4%) | 105 (13%) | 174 (9.3%) |  | 103 (7.7%) | 176 (13.3%) |  | 6 (9%) | 273 (10.5%) |  |
| Somewhat agree | 510(19.2%) | 133 (16.5%) | 377 (20.3%) |  | 277 (20.7%) | 233 (17.6%) |  | 8 (11.9%) | 502 (19.3%) |  |
| Agree | 1516(56.9%) | 411 (51.1%) | 1105 (59.5%) |  | 803 (60%) | 713 (53.9%) |  | 37 (55.2%) | 1479 (57%) |  |

Note: For items 1-11, see Table 1. Fisher’s exact test was used in the analysis of sizes (<5).

**Appendix S2**. Demographic characteristics (age, education level, and relatives or friends living in COVID-19 epicentre) of participants (N=2263)

| Option | Age (years) | | | Education level | | | | Relatives or friends living  in COVID-19 epicentre | | | |  |
| --- | --- | --- | --- | --- | --- | --- | --- | --- | --- | --- | --- | --- |
|  | 18-45  (N=2073) | 46-59  (N=555) | ≥60  (N=35) | | 1  (N=430) | 2  (N=1404) | 3  (N=829) | | Yes  (N=356) | No  (N=2273) | Not sure  (N=34) | |
| **Awareness of COVID-19** |  |  |  | |  |  |  | |  |  |  | |
| 1 Before Dec 28^th^, 2019 | 574 (27.7%) | 133 (24%) | 11 (31.4%) | | 106 (24.6%) | 373 (26.6%) | 239 (28.8%) | | 113 (31.7%) | 595 (26.2%) | 10 (29.4%) | |
| Adjusted residuals | 1.6 | -1.8 | 0.6 | | -1.2 | -0.5 | 1.5 | | 2.2 | -2.2 | 0.3 | |
| Before Jan 17^th^, 2020 | 675 (32.6%) | 168 (30.2%) | 9 (25.7%) | | 107 (24.9%) | 447 (31.8%) | 298 (35.9%) | | 120 (33.7%) | 721 (31.7%) | 11 (32.4%) | |
| Adjusted residuals | 1.2 | -1.0 | -0.8 | | **-3.5** | -0.2 | 2.9 | | 0.7 | -0.7 | 0 | |
| Before Jan 20^th^, 2020 | 658 (31.7%) | 184 (33.2%) | 13 (37.1%) | | 153 (35.5%) | 455 (32.4%) | 247 (29.8%) | | 101 (28.4%) | 743 (32.7%) | 11 (32.4%) | |
| Adjusted residuals | -0.8 | 0.6 | 0.6 | | 1.7 | 0.4 | -1.7 | | -1.6 | 1.6 | 0 | |
| Before Jan 23^rd^, 2020 | 166 (8%) | 70 (12.6%) | 2 (5.8%) | | 64 (15%) | 129 (9.2%) | 45 (5.5%) | | 22 (6.2%) | 214 (9.4%) | 2 (5.8%) | |
| Adjusted residuals | **-3.2** | **3.4** | -0.7 | | **4.7** | 0.5 | **-4.3** | | -2.0 | 2.1 | -0.6 | |
| 2 Not sure | 202 (9.7%) | 50 (9%) | 3 (8.5%) | | 47 (10.9%) | 122 (8.7%) | 86 (10.4%) | | 33 (9.3%) | 219 (9.6%) | 3 (8.8%) | |
| Adjusted residuals | 0.6 | -0.5 | -0.2 | | 1.0 | -1.6 | 0.9 | | -0.2 | 0.3 | -0.2 | |
| Mild | 538 (26%) | 120 (21.6%) | 8 (22.8%) | | 81 (18.8%) | 341 (24.3%) | 244 (29.4%) | | 102 (28.7%) | 555 (24.4%) | 9 (26.5%) | |
| Adjusted residuals | 2.1 | -2.1 | -0.3 | | **-3.2** | -0.9 | **3.5** | | 1.7 | -1.7 | 0.2 | |
| Moderate | 691 (33.3%) | 220 (39.6%) | 15 (43%) | | 148 (34.4%) | 503 (35.8%) | 275 (33.2%) | | 116 (32.6%) | 803 (35.4%) | 7 (20.6%) | |
| Adjusted residuals | -2.9 | 2.7 | 1.0 | | -0.2 | 1.2 | -1.2 | | -0.9 | 1.5 | -1.7 | |
| Severe | 642 (31%) | 165 (29.8%) | 9 (25.7%) | | 154 (35.9%) | 438 (31.2%) | 224 (27%) | | 105 (29.4%) | 696 (30.6%) | 15 (44.1%) | |
| Adjusted residuals | 0.7 | -0.5 | -0.6 | | 2.5 | 0.7 | -2.7 | | -0.5 | -0.1 | 1.7 | |
| 3 Every 3 days | 70 (3.4%) | 30 (5.4%) | 1 (2.8%) | | 14 (3.2%) | 46 (3.2%) | 41 (4.9%) | | 13 (3.7%) | 87 (3.8%) | 1 (2.9%) | |
| Adjusted residuals | -2.1 | 2.2 | -0.3 | | -0.6 | -1.5 | 2.1 | | -0.1 | 0.2 | -0.3 | |
| Every day | 590 (28.5%) | 173 (31.2%) | 11 (31.4%) | | 134 (31.1%) | 419 (29.8%) | 221 (26.7%) | | 102 (28.7%) | 663 (29.2%) | 9 (26.5%) | |
| Adjusted residuals | -1.3 | 1.2 | 0.3 | | 1.0 | 0.9 | -1.8 | | -0.2 | 0.3 | -0.3 | |
| Every half day | 508 (24.5%) | 135 (24.3%) | 12 (34.4%) | | 115 (26.8%) | 350 (25%) | 190 (22.9%) | | 71 (19.9%) | 574 (25.3%) | 10 (29.4%) | |
| Adjusted residuals | -0.2 | -0.2 | 1.3 | | 1.1 | 0.4 | -1.4 | | -2.2 | 1.9 | 0.7 | |
| It depends | 905 (43.6%) | 217 (39.1%) | 11 (31.4%) | | 167 (38.9%) | 589 (42%) | 377 (45.5%) | | 170 (47.7%) | 949 (41.7%) | 14 (41.2%) | |
| Adjusted residuals | 2.2 | -1.8 | -1.3 | | -1.7 | -0.7 | 2.1 | | 2.1 | -2.0 | -0.2 | |
| 4 Not sure | 614 (29.6%) | 122 (22%) | 10 (28.6%) | | 155 (36%) | 405 (28.8%) | 186 (22.4%) | | 86 (24.2%) | 645 (28.4%) | 15 (44.1%) | |
| Adjusted residuals | **3.5** | **-3.6** | 0.1 | | **4.1** | 1.0 | **-4.3** | | -1.7 | 1.0 | 2.1 | |
| Feb 8^th^ | 179 (8.7%) | 81 (14.5%) | 6 (17.1%) | | 51 (11.8%) | 153 (11%) | 62 (7.5%) | | 19 (5.3%) | 243 (10.7%) | 4 (11.8%) | |
| Adjusted residuals | **-4.4** | **4.1** | 1.4 | | 1.4 | 1.7 | -2.9 | | **-3.1** | 2.9 | 0.3 | |
| The end of February | 938 (45.2%) | 272 (49%) | 19 (54.3%) | | 165 (38.4%) | 648 (46.1%) | 416 (50.2%) | | 183 (51.4%) | 1036 (45.6%) | 10 (29.4%) | |
| Adjusted residuals | -1.8 | 1.5 | 1.0 | | **-3.5** | 0 | 2.8 | | 2.1 | -1.4 | -2.0 | |
| The end of May | 342 (16.5%) | 80 (14.5%) | 0 (0%) | | 59 (13.8%) | 198 (14.1%) | 165 (19.9%) | | 68 (19.1%) | 349 (15.3%) | 5 (14.7%) | |
| Adjusted residuals | 1.7 | -1.0 | -2.6 | | -1.3 | -2.5 | **3.9** | | 1.8 | -1.7 | -0.2 | |
| **Response towards COVID-19** |  |  |  | |  |  |  | |  |  |  | |
| 5 Do nothing | 102 (4.9%) | 61 (11%) | 5 (14.3%) | | 22 (5.1%) | 75 (5.3%) | 71 (8.5%) | | 25 (7%) | 142 (6.2%) | 1 (2.9%) | |
| Adjusted residuals | **-5.5** | **5.1** | 2.0 | | -1.1 | -2.2 | **3.2** | | 0.6 | -0.3 | -0.8 | |
| Inform others | 1559 (75.2%) | 333 (60%) | 24 (68.6%) | | 310 (72.1%) | 1043 (74.3%) | 563 (67.9%) | | 254 (71.3%) | 1637 (72%) | 25 (73.6%) | |
| Adjusted residuals | **7.0** | **-7.0** | -0.4 | | 0.1 | 2.8 | **-3.1** | | -0.3 | 0.2 | 0.2 | |
| Prepare supplies | 330 (15.9%) | 97 (17.4%) | 6 (17.1%) | | 80 (18.6%) | 204 (14.5%) | 149 (18%) | | 65 (18.3%) | 361 (15.9%) | 7 (20.6%) | |
| Adjusted residuals | -0.9 | 0.9 | 0.1 | | 1.4 | -2.6 | 1.6 | | 1.1 | -1.3 | 0.7 | |
| Fight against COVID-19 | 82 (4%) | 64 (11.6%) | 0 (0%) | | 18 (4.2%) | 82 (5.9%) | 46 (5.6%) | | 12 (3.4%) | 133 (5.9%) | 1 (2.9%) | |
| Adjusted residuals | **-6.5** | **7.0** | -1.4 | | -1.3 | 0.9 | 0.1 | | -1.9 | 2.0 | -0.7 | |
| 6 Walk or change transportation | 17 (0.8%) | 8 (1.4%) | 0 (0%) | | 4 (0.9%) | 12 (0.9%) | 9 (1.1%) | | 5 (1.4%) | 20 (0.9%) | 0 (0%) | |
| Adjusted residuals | -1.2 | 1.4 | -0.6 | | 0 | -0.5 | 0.5 | | 1.0 | -0.8 | -0.6 | |
| Cover your nose and mouth with your sleeves | 78 (3.8%) | 33 (5.9%) | 3 (8.6%) | | 26 (6.1%) | 54 (3.8%) | 34 (4.1%) | | 14 (3.9%) | 98 (4.3%) | 2 (5.8%) | |
| Adjusted residuals | -2.5 | 2.2 | 1.3 | | 2.0 | -1.2 | -0.3 | | -0.3 | 0.2 | 0.5 | |
| Ignore | 1223 (59%) | 269 (48.5%) | 13 (37.1%) | | 238 (55.3%) | 825 (58.8%) | 442 (53.3%) | | 205 (57.6%) | 1273 (56%) | 27 (79.5%) | |
| Adjusted residuals | **4.8** | **-4.3** | -2.3 | | -0.5 | 2.5 | -2.2 | | 0.4 | -1.3 | 2.7 | |
| It depends | 755 (36.4%) | 245 (44.2%) | 19 (54.3%) | | 162 (37.7%) | 513 (36.5%) | 344 (41.5%) | | 132 (37.1%) | 882 (38.8%) | 5 (14.7%) | |
| Adjusted residuals | **-3.7** | **3.2** | 2.0 | | -0.3 | -1.9 | 2.3 | | -0.5 | 1.4 | -2.8 | |
| **Perception and attitude towards COVID-19** |  |  |  | |  |  |  | |  |  |  | |
| 7 Disagree | 8 (0.3%) | 8 (1.4%) | 0 (0%) | | 6 (1.4%) | 2 (0.1%) | 8 (0.9%) | | 2 (0.6%) | 13 (0.6%) | 1 (2.9%) | |
| Adjusted residuals | -2.7 | 2.9 | -0.5 | | 2.3 | **-3.2** | 1.6 | | -0.1 | -0.5 | 1.8 | |
| Not sure | 23 (1.2%) | 14 (2.6%) | 1 (2.8%) | | 5 (1.1%) | 20 (1.4%) | 13 (1.6%) | | 13 (3.7%) | 25 (1.1%) | 0 (0%) | |
| Adjusted residuals | -2.6 | 2.4 | 0.7 | | -0.5 | 0 | 0.4 | | **3.8** | **-3.4** | -0.7 | |
| Somewhat agree | 723 (34.8%) | 191 (34.4%) | 10 (28.6%) | | 116 (27%) | 473 (33.7%) | 335 (40.4%) | | 149 (41.8%) | 765 (33.7%) | 10 (29.4%) | |
| Adjusted residuals | 0.4 | -0.2 | -0.8 | | **-3.7** | -1.2 | **4.2** | | 3.0 | -2.7 | -0.7 | |
| Agree | 1319 (63.7%) | 342 (61.6%) | 24 (68.6%) | | 303 (70.5%) | 909 (64.8%) | 473 (57.1%) | | 192 (53.9%) | 1470 (64.6%) | 23 (67.7%) | |
| Adjusted residuals | 0.7 | -0.9 | 0.7 | | **3.4** | 1.7 | **-4.5** | | **-3.9** | **3.6** | 0.5 | |
| 8 Disagree | 7 (0.3%) | 4 (0.7%) | 0 (0%) | | 3 (0.7%) | 5 (0.3%) | 3 (0.4%) | | 2 (0.6%) | 9 (0.4%) | 0 (0%) | |
| Adjusted residuals | -1.1 | 1.3 | -0.4 | | 1.0 | -0.5 | -0.3 | | 0.5 | -0.3 | -0.4 | |
| Not sure | 81 (3.9%) | 11 (2%) | 1 (2.9%) | | 10 (2.3%) | 47 (3.4%) | 36 (4.3%) | | 19 (5.3%) | 72 (3.2%) | 2 (5.8%) | |
| Adjusted residuals | 2.2 | -2.2 | -0.2 | | -1.4 | -0.4 | 1.6 | | 2.0 | -2.2 | 0.8 | |
| Somewhat agree | 59 (2.9%) | 18 (3.2%) | 4 (11.4%) | | 12 (2.8%) | 32 (2.2%) | 37 (4.5%) | | 17 (4.8%) | 63 (2.8%) | 1 (2.9%) | |
| Adjusted residuals | -1.1 | 0.3 | 2.9 | | -0.3 | -2.4 | 2.9 | | 2.0 | -2.0 | 0 | |
| Agree | 1926 (92.9%) | 522 (94.1%) | 30 (85.7%) | | 405 (94.2%) | 1320 (94.1%) | 753 (90.8%) | | 318 (89.3%) | 2129 (93.6%) | 31 (91.3%) | |
| Adjusted residuals | -0.5 | 1.0 | -1.7 | | 1.0 | 2.1 | -3.0 | | -3.0 | 3.0 | -0.4 | |
| 9 Disagree | 45 (2.2%) | 32 (5.8%) | 0 (0%) | | 13 (3%) | 32 (2.3%) | 32 (3.8%) | | 13 (3.6%) | 62 (2.7%) | 2 (5.8%) | |
| Adjusted residuals | **-4.2** | **4.5** | -1.0 | | 0.2 | -2.0 | 2.0 | | 0.9 | -1.2 | 1.0 | |
| Not sure | 4 (0.1%) | 7 (1.2%) | 0 (0%) | | 4 (0.9%) | 4 (0.3%) | 3 (0.4%) | | 3 (0.8%) | 8 (0.4%) | 0 (0%) | |
| Adjusted residuals | **-3.3** | **3.5** | -0.4 | | 1.8 | -1.1 | -0.3 | | 1.4 | -1.2 | -0.4 | |
| Somewhat agree | 924 (44.6%) | 223 (40.2%) | 14 (40%) | | 144 (33.5%) | 601 (42.8%) | 416 (50.2%) | | 170 (47.8%) | 978 (43%) | 13 (38.3%) | |
| Adjusted residuals | 1.9 | -1.8 | -0.4 | | **-4.6** | -0.9 | **4.6** | | 1.7 | -1.4 | -0.6 | |
| Agree | 1100 (53.1%) | 293 (52.8%) | 21 (60%) | | 269 (62.6%) | 767 (54.6%) | 378 (45.6%) | | 170 (47.8%) | 1225 (53.9%) | 19 (55.9%) | |
| Adjusted residuals | -0.1 | -0.2 | 0.8 | | **4.3** | 1.7 | **-5.2** | | -2.2 | 2.0 | 0.3 | |
| 10 Disagree | 9 (0.4%) | 2 (0.3%) | 0 (0%) | | 4 (0.9%) | 4 (0.3%) | 3 (0.4%) | | 3 (0.8%) | 8 (0.4%) | 0 (0%) | |
| Adjusted residuals | 0.3 | -0.2 | -0.4 | | 1.8 | -1.1 | -0.3 | | 1.4 | -1.2 | -0.4 | |
| Not sure | 15 (0.7%) | 3 (0.5%) | 0 (0%) | | 5 (1.1%) | 6 (0.4%) | 7 (0.8%) | | 5 (1.4%) | 12 (0.5%) | 1 (2.9%) | |
| Adjusted residuals | 0.6 | -0.4 | -0.5 | | 1.3 | -1.7 | 0.7 | | 1.8 | -2.3 | 1.6 | |
| Somewhat agree | 25 (1.2%) | 4 (0.8%) | 0 (0%) | | 1 (0.2%) | 21 (1.5%) | 7 (0.8%) | | 12 (3.4%) | 16 (0.7%) | 1 (2.9%) | |
| Adjusted residuals | 1.1 | -0.9 | -0.6 | | -1.9 | 2.1 | -0.8 | | **4.5** | **-4.6** | 1.0 | |
| Agree | 2024 (97.7%) | 546 (98.4%) | 35 (100%) | | 420 (97.8%) | 1373 (97.8%) | 812 (98%) | | 336 (94.4%) | 2237 (98.4%) | 32 (94.2%) | |
| Adjusted residuals | -1.2 | 1.0 | 0.9 | | -0.2 | -0.1 | 0.3 | | **-4.8** | **5.1** | -1.5 | |
| 11 Disagree | 270 (13%) | 83 (15%) | 5 (14.3%) | | 36 (8.3%) | 175 (12.4%) | 147 (17.7%) | | 66 (18.5%) | 287 (12.6%) | 5 (14.7%) | |
| Adjusted residuals | -1.2 | 1.2 | 0.1 | | **-3.4** | -1.6 | **4.4** | | 3.0 | -3.0 | 0.2 | |
| Not sure | 212 (10.2%) | 65 (11.7%) | 2 (5.7%) | | 27 (6.3%) | 129 (9.2%) | 123 (14.8%) | | 44 (12.4%) | 228 (10%) | 7 (20.6%) | |
| Adjusted residuals | -0.8 | 1.1 | -0.9 | | **-3.1** | -2.3 | **4.9** | | 1.2 | -1.8 | 1.9 | |
| Somewhat agree | 403 (19.4%) | 100 (18%) | 7 (20%) | | 74 (17.2%) | 257 (18.3%) | 179 (21.6%) | | 70 (19.7%) | 433 (19%) | 7 (20.6%) | |
| Adjusted residuals | 0.7 | -0.8 | 0.1 | | -1.1 | -1.2 | 2.2 | | 0.3 | -0.3 | 0.2 | |
| Agree | 1188 (57.4%) | 307 (55.3%) | 21 (60%) | | 293 (68.2%) | 843 (60.1%) | 380 (45.9%) | | 176 (49.4%) | 1325 (58.4%) | 15 (44.1%) | |
| Adjusted residuals | 0.7 | -0.9 | 0.4 | | **5.1** | **3.4** | **-7.8** | | **-3.1** | **3.4** | -1.5 | |

Note: For items 1-11, see Table 1. Fisher’s exact test was used in the analysis of sizes (<5). Education level 1 = lower than undergraduate, Education level 2 = undergraduate, Education level 3 = postgraduate
